# Evaluation of Saffron Quality Using Rapid Quantitative Inspection Technology with Near-Infrared Spectroscopy

**DOI:** 10.3390/molecules29173983

**Published:** 2024-08-23

**Authors:** Ying Zhou, Han Zhang, Xiaohui Sheng, Rong Wang, Yao Yao, Qinglan Zhu, Ze Yi, Zhe Xu, Yi Wang, Cheng Zheng, Yu Tang

**Affiliations:** 1 NMPA Key Laboratory of Quality Evaluation of Traditional Chinese Medicine (Traditional Chinese Patent Medicine), Zhejiang Institute for Food and Drug Control, Hangzhou 310052, China; zyayd@126.com (Y.Z.); wangrong017@163.com (R.W.); yaoyao@zjyj.org.cn (Y.Y.); zhuqinglan@zjyj.org.cn (Q.Z.); cpu_izaya@163.com (Z.Y.); 2 Pharmaceutical Informatics Institute, College of Pharmaceutical Sciences, Zhejiang University, Hangzhou 310058, China; 11919014@zju.edu.cn (H.Z.); zjuwangyi@zju.edu.cn (Y.W.); 3 Hangzhou Puyu Technology Development Co., Ltd., Hangzhou 310052, China; xiaohui_heng@fpi-inc.com; 4 College of Pharmacy, Shenyang Pharmaceutical University, Benxi 117004, China; 13630902846@163.com

**Keywords:** saffron, near-infrared spectroscopy, quantitative models, rapid quality evaluation system

## Abstract

A predictive model utilizing near-infrared spectroscopy was developed to estimate the loss on drying, total contents of crocin I and crocin II, and picrocrocin content of saffron. Initially, the LD values were determined using a moisture-ash analyzer, while HPLC was employed for measuring the total contents of crocin I, crocin II, and picrocrocin. The near-infrared spectra of 928 saffron samples were collected and preprocessed using first derivative, standard normal variable transformation, detrended correction, multivariate scattering correction, Savitzky–Golay smoothing, and mean centering methods. Leveraging the partial least squares method, regression models were constructed, with parameters optimized through a selective combination of the above six preprocessing methods. Subsequently, prediction models for loss on drying, total contents of crocin I and crocin II, and picrocrocin content were established, and the prediction accuracy of the models was verified. The correlation coefficients and root mean square error of loss on drying, total contents of crocin I and crocin II, and picrocrocin content demonstrated high accuracy, with *R*^2^ values of 0.8627, 0.8851, and 0.8592 and root mean square error values of 0.0260, 0.0682, and 0.0465. This near-infrared prediction model established in the present study offers a precise and efficient means of assessing loss on drying, total contents of crocin I and crocin II, and picrocrocin content in saffron and is useful for the development of a rapid quality evaluation system.

## 1. Introduction

Saffron, which is the dried stigmas of *Crocus sativus* L. of the Iridaceae family, is a traditional spice widely used around the world. It is also well known in China as a medicinal herb, utilized to promote blood circulation, reduce bruising, lower blood sugar levels, and provide antioxidant and antitumor benefits. Saffron is listed in the *Chinese Pharmacopoeia*, the *European Pharmacopoeia*, the *British Pharmacopoeia*, and other national standards [1,2,3,4].

Iran is the world’s leading saffron producer, and China imports a substantial amount of saffron from Iran. In recent years, the increased planting area of saffron in Iran has led to a significant expansion in the scales of customs declarations and inspections of saffron in China. Imports have surged from less than 400 kilograms in 2014 to nearly 20 tons in 2020. Data regarding the import of saffron between 2020 and 2023 are currently not available due to the impact of the COVID-19 pandemic. This explosive growth has resulted in large quantities of saffron requiring testing according to the *Chinese Pharmacopoeia* by port inspection centers. Consequently, this has severely limited the timeliness of detection and quality control while significantly increasing inspection costs. Although the methods prescribed by the *Chinese Pharmacopoeia* are highly sensitive and accurate, they involve the use of toxic chemicals for sample preparation, damage the samples, and require a long time for analysis [1]. These methods are unsuitable for rapid saffron analysis, highlighting the need for a safe, fast, and reliable alternative. Near-infrared light, an electromagnetic wave with a wavelength range of 780–2526 nm, primarily reflects the overtone and combination frequency absorption of hydrogen-containing groups (such as C-H, N-H, S-H, and O-H). This interaction provides detailed structural and compositional information about a sample. On the other hand, with near-infrared spectroscopy (NIR), sample preparation is simple, the cost is low, and analysis is simple. Collectively, NIR is a comprehensive analytical technology that enables nondestructive, rapid, quantitative analysis [5,6,7,8].

The quality of saffron, as a traditional medicinal herb, is primarily determined by loss on drying (LD), total contents of crocin I (C_44_H_64_O_24_) and crocin II (C_38_H_54_O_19_) (TCCC), and picrocrocin (C_16_H_26_O_7_) content (CP). According to the *Chinese Pharmacopoeia*, these three aspects are used as key indices for assessing saffron quality. Basically, the LD should not exceed 12.0%, the TCCC should be at least 10.0%, and the CP should be no less than 5.0%. Owing to the wide use of saffron, a wealth of studies have been performed to evaluate the quality of this herb, including the use of physical checks, chemical methods, and molecular methods. However, most of these methods or techniques are laborious and/or not sufficiently precise [9]. In addition, the commonly used spectral methods need reference standards and destroy the samples tested [9].

A comprehensive literature survey revealed that NIR has been used to investigate the chemical compositions of polysaccharides and oils, moisture and loureirin contents, and other quality indices [10,11,12,13,14,15,16,17]. It has also been employed to explore the authenticity and identify the species of Chinese medicinal materials, such as for the rapid detection of adulterants in *Dendrobium huoshanense* and for the determination of moisture content in honey-processed Asteris Radix et Rhizoma [18,19,20,21,22,23]. In summary, NIR can serve as a valuable tool for detecting adulteration and for the routine quality control of herbal materials with complex matrices. However, most NIR studies on saffron, both domestically and internationally, have focused on authenticity identification, with few addressing overall quality management. To save time, reduce costs, and improve the efficiency of saffron detection, the main goal of this study was to establish and calibrate quantitative NIR models using partial least squares (PLS). These models were used to rapidly determine quality indices using data from a near-infrared analyzer measuring the LD and from high-performance liquid chromatography (HPLC) experiments determining the TCCC and CP. This approach is crucial for developing a quality evaluation system for the rapid assessment of saffron.

## 2. Results and Discussion

### 2.1. Statistical Data Used for Predictive Modeling

**Figure 1:**
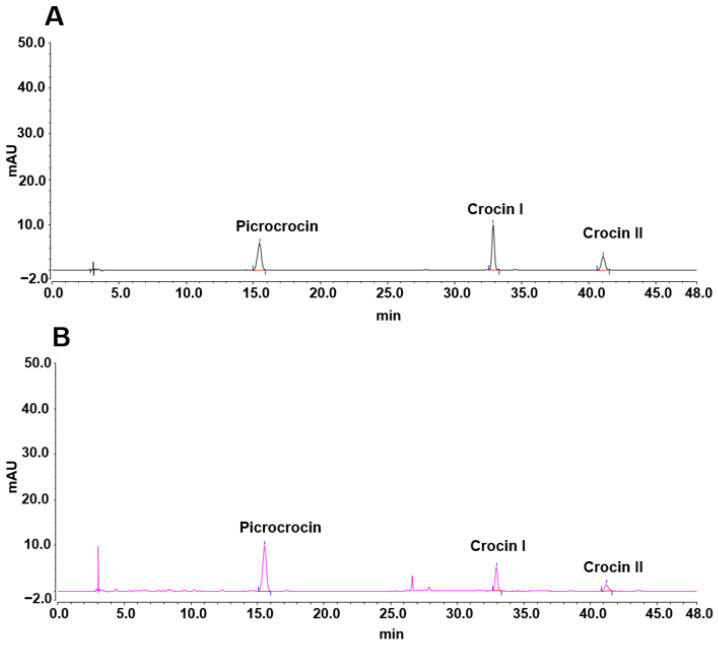
Representative chromatograms of saffron and standard substances in saffron. (**A**) Chromatogram of picrocrocin, crocin I, and crocin II; (**B**) chromatogram of saffron.

**Figure 2:**
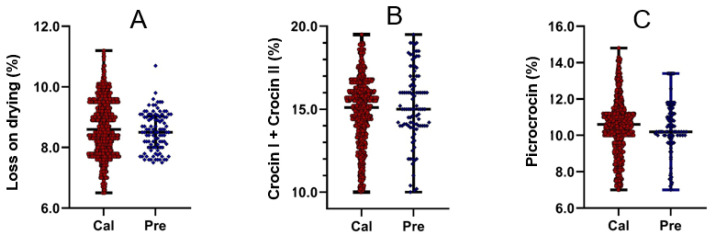
Distribution ranges of LD (**A**), TCCC (**B**), and CP (**C**) in the saffron samples.

It is well known that a key consideration in establishing a prediction model is the diversity of data used for modeling, as the accuracy of a model increases with the amount and type of data used. A calibration set with a wide coverage range can improve the accuracy and stability of the model being established. In this research, a total of 828 saffron samples were randomly selected as the calibration set for the prediction model, with the remaining 100 samples used as the verification set. The current edition of the *Chinese Pharmacopoeia* states that “the TCCC should not be less than 10.0% and the CP should not be less than 5.0%”. Additionally, the concentrations of these three molecules in saffron determine its quality and commercial value [24]. Therefore, picrocrocin and the sum of the crocin I and II contents were used as indices in this investigation to align with the detection standards of the *Chinese Pharmacopoeia*. Additionally, large amounts of water or other volatile substances in a drug not only reduce the drug’s purity and affect the dosage accuracy but also increase the risk of hydrolysis or spoilage, potentially leading to substandard therapeutic effects. Thus, measuring the LD is essential. First of all, according to the method recorded in the *Chinese Pharmacopoeia*, the HPLC profiles of saffron and its standard substances, namely, crocin I, crocin II, and picrocrocin, were established, as shown in Figure 1. The chromatograms of the reference standards and the saffron samples exhibited excellent congruence in terms of retention time, indicating that the HPLC results for the saffron samples qualified for the subsequent determination of the contents of the selected compounds. The contents of the standard substances were therefore calculated (Table 1), and the typical heating program map for the LD and its values are shown in Figure S1. Collectively, the data regarding the content ranges of the standard substances and the LD of the saffron samples are shown in Table 1 and Figure 2, where the content ranges of the LD, TCCC, and CP on the calibration set were 6.4–11.2%, 10.0–19.5%, and 7.0–14.8%, respectively. In general, the data selected were inclusive and suitable for model building, which could further enable the rapid and accurate determination of those key factors for the evaluation of saffron quality.

### 2.2. Original Near-Infrared Spectra of Saffron

**Figure 3:**
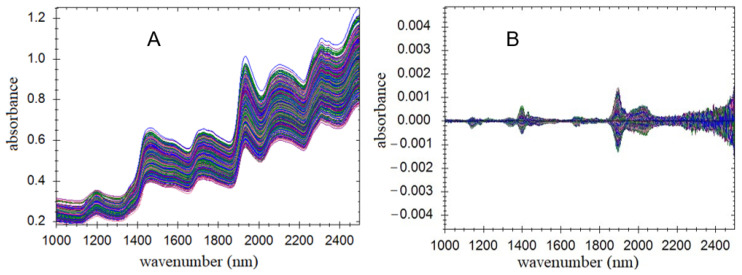
Original (**A**) and pretreated (**B**) NIR spectra for predictive modeling.

The NIR spectra of the 928 saffron samples are shown in Figure 3. Generally, the original NIR spectra (Figure 3A) showed similar trends and exhibited strong absorptions at around 1200, 1450, 1700, 1950, 2100, and 2300 nm, with two weaker absorption peaks at around 1380 and 1580 nm. The absorption peaks appearing at around 1200 and 1400 nm were primarily due to the stretching vibrations of C-H and O-H bonds. The peaks at 2100 and 2300 nm mainly originated from the first-order overtone of the methyl group (CH_3_), the combined frequency of C-H bonds in sugars, and the stretching vibrations of O-H bonds [25]. Although the spectral trends of the different saffron samples were consistent, differences assignable to a small number of samples in the 1050–2400 nm region were observed, which indicated chemical discrepancies among the samples. Notably, the sampling time for a single saffron sample was usually around 1 min, demonstrating the speed of NIR as a method for this purpose.

### 2.3. Division of Sample Set

A total of 928 samples were partitioned, with 100 allocated for verification, and 8-fold cross-validation was employed for analysis. The exclusion of abnormal samples was conducted, and the performance of each model on the cross-validation set was as presented in Table 2.

### 2.4. Spectral Data Preprocessing

The original spectral data were processed by applying a selected combination of six preprocessing approaches before predictive modeling: first derivative (FD), standard normal variable transformation (SNV), detrended correction (DT), multivariate scattering correction (MSC), Savitzky–Golay smoothing, and mean centering. Then, a PLS model was established after the processing of the original spectra using methods 1, 2, and 3, and the resultant outcomes are shown in Table 2 (see the Supporting Information for an explanation of each method’s impact on the model’s performance). In addition, the processed NIR spectra derived from the original ones are shown in Figure 3B. Based on the results in Table 2, the optimal combination of spectral preprocessing algorithms for the TCCC, CP, and LD near-infrared correction model was found to be method 2, which included MSC + Savitzky–Golay smoothing + first-derivative Savitzky–Golay filtering + mean centering. In the model based on method 2, the *R* values of the calibration set for the LD, TCCC and CP were 0.96, 0.90, and 0.91, respectively, while the SECV values were 0.2763, 0.9859, and 0.6836 for each parameter. The high *R* values and low SEC and SECV values indicated that the model was capable of producing accurate predictions, with RPD (Equation (1)) values being 3.571, 2.294, and 2.412, respectively.

(1) $$RPD=\frac{1}{\sqrt{1-R^{2}}}$$

### 2.5. Model Verification and Evaluation

**Figure 4:**
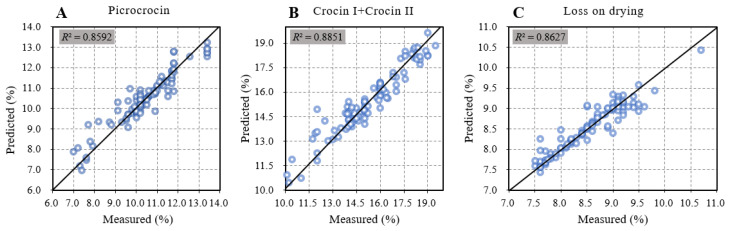
Linear regression of the test set samples. (**A**):TCCC; (**B**) CP; (**C**) LD.

To further verify the performance of the near-infrared calibration model, the TCCC, CP, and LD of 100 independent saffron samples were predicted using the PLS model. As shown in Figure 4, the experimental values (Tables S1–S3, see the Supporting Information) of the TCCC, CP, and LD of the saffron samples were in good agreement with the predicted ones with *R*² values of 0.8851 for the TCCC, 0.8592 for the CP, and 0.8627 for the LD. Moreover, a paired sample T-test was conducted with a confidence level of 95% to corroborate the results generated by the prediction model. The *p*-values obtained from the T-test for the TCCC, CP, and LD were 0.953, 0.942, and 0.942, respectively, indicating no significant differences between the predicted and measured data. In summary, the established prediction model can accurately predict the contents of the components of saffron, enabling the confident evaluation of saffron samples quality.

## 3. Materials and Methods

### 3.1. Chemicals and Reagents

Crocin I [batch number (BN):111588-201704, mass fraction (MF): 88.4%], crocin II (BN:111588-201705, MF: 92.2%), and picrocrocin (BN: 112056-202001, MF: 97.6%) reference substances were all purchased from the China Institute for Food and Drug Control; acetonitrile was HPLC-grade (Merck & Co., Rahway, NJ, USA); ethanol was analytical pure; ultrapure water was prepared using a Milli-Q system.

### 3.2. Screening of the Chromatographic Elution Program

The HPLC analysis was performed on a Dionex UltiMate 3000 LC series diode array detector (DAD) system with a quaternary pump (thermo) and an autosampler that could thermostat samples. Separation was achieved on an Ultimate Plus C_18_ column (4.6 mm × 250 mm, 5 µm, Welch Materials, Inc., West Haven, CT, USA). The detection wavelength was set to 440 nm for crocin I and II and to 254 nm for picrocrocin. The injection volume was 10 µL.

Chromatographic separation was performed using acetonitrile (solvent A) and water (solvent B) as the mobile phase at a flow rate of 1 mL/min. The gradient program was set as follows: 0–20 min: isocratic 13% A; 20–23 min: 13% A–23% A; 23–45 min: 23% A–25% A; 45–50 min: 25% A–50% A.

Samples were quantified via the external standard method using their peak areas. The HPLC results were used for reference in the NIR spectroscopy analysis (Hangzhou Puyu Technology Inc., Hangzhou, China).

### 3.3. Saffron Sample Collection and Processing

Saffron samples from Iran (numbered S1 to S928) were obtained directly from Hangzhou Customs Inspection in China. Among these, 40 samples were collected in 2020, 456 samples in 2021, and 432 samples in 2022. Throughout the three years of sample collection, the related testing in this study was carried out according to the legal standards, and the near-infrared rapid detection of saffron was conducted simultaneously. As a result, the model underwent continuous training over the three years, ultimately performing prediction with high accuracy. After sampling, the saffron samples were deposited at the Herbarium of NMPA Key Laboratory of Quality Evaluation of Traditional Chinese Medicine (Traditional Chinese Patent Medicine), Zhejiang Institute for Food and Drug Control.

The samples were placed at room temperature for 2 h before experimentation. The software required a 30 min preheat period after the instrument was powered on. Subsequently, the samples were placed in the sample tray for scanning and spectral information collection. A sample of 10 g was prepared for NIR spectroscopy. A NIR spectrometer (Hangzhou Puyu Technology Inc., China) scanned samples from 1000 to 2499 nm, using a circular sample cup (100 mm in diameter, 20 mm in height). Data were saved as the average of 30 scans.

### 3.4. LD Experiment with Saffron Samples

The LD of the saffron samples was measured following the method outlined in the *Pharmacopeia of the People’s Republic of China* (Volume IV 0831, 2020 edition). For LD determination, the saffron materials were thoroughly mixed, and 2 g of the mixed sample was weighed. The weighed sample was placed in a tared, shallow weighing bottle that was previously dried to a constant weight at 105 °C. Finally, the sample was dried at 105 °C for 6 h, and the percentage of LD was calculated accordingly.

### 3.5. TCCC and CP Determination 

TCCC and CP were measured following the method outlined in the *Pharmacopeia of the People’s Republic of China* (Volume I, 2020 edition). For TCCC and CP determination, 10 mg of powdered sample was accurately weighed and placed in 50 mL of 50% ethanol in a dark brown volumetric flask. The sample was ultrasonically extracted in an ice bath for 20 min. After standing at room temperature, the volume was adjusted with 50% ethanol. The extracts were filtered through a 0.22 μm nylon syringe filter (Jinkong, Tianjin Keyilong Lab Equipment Co., Ltd., Tianjin, China) and analyzed using a Dionex UltiMate 3000 LC series diode array detector (DAD) system with a quaternary pump.

### 3.6. Statistical Analysis

Statistical analysis was performed using RIMP software (version 14.0, Hangzhou Puyu Technology Inc., China), which was used for spectra treatments and calibration development. Spectra were mathematically corrected for light scattering using multiple scattering correction (MSC), Savitzky–Golay smoothing, first derivative Savitzky–Golay filter, and mean centering. Paired sample *T*-tests were conducted with SPSS software (version 18).

### 3.7. Near-Infrared Spectra Preprocessing Methods 

The accuracy of a near-infrared model can be affected by noise, instrument-to-instrument variation, and scattered light in the environment. Pretreatment of the collected near-infrared spectra can mitigate the effects of these interferences. The near-infrared spectral information of saffron was obtained from a near-infrared analyzer. A partial least squares (PLS) model was established after preprocessing the spectral data. The preprocessing methods used were standard normal variate (SNV) transformation, detrended correction (DT), multiple scattering correction (MSC), Savitzky–Golay smoothing, first-derivative Savitzky–Golay filtering, and mean centering. Abnormal samples were ruled out using a studentized residual plot, and the final model was established using the partial least squares method.

### 3.8. Selection of Characteristic Spectral Variables

Utilizing full-spectrum modeling inevitably includes measuring noise bands unrelated to the components. Removing noise wavelengths serves to simplify the model and eliminate irrelevant spectral variables, thereby enhancing the prediction accuracy and stability of the calibrated model. For these reasons, we excluded the extremities and ultimately selected the spectral range of 1050–2400 nm as the characteristic band for modeling.

### 3.9. Modeling Methods and Model Evaluation

This approach involved using a studentized residual plot to identify and remove abnormal samples. The model was evaluated using the correlation coefficient (*R*), standard error of calibration (SEC), standard error of cross-validation (SECV), and the ratio of performance to deviation (RPD). A reliable model is indicated when the SECV is not more than 1.2 times the SEC, *R* approaches 1, and RPD ranges between 1.4 and 2.0. An RPD value exceeding 2.0 demonstrates optimal prediction ability and model stability.

## 4. Conclusions

In this study, to address the ever-increasing demand for the inspection of imported saffron, a prediction model involving a NIR technique was developed for the rapid and quantitative analysis of the LD, TCCC, and CP of saffron samples. The results generated by the established model were compared with those obtained from traditional pharmacopeia analytical methods, which indicated that the prediction model could provide reliable results for evaluating the quality of saffron. Of note, the combination of MSC, Savitzky–Golay smoothing, Savitzky–Golay first-derivative filtering, and mean centering yielded the optimal model for the determination of LD, TCCC, and CP with high confidence. Moreover, the combination can be applied directly in the context of evaluating saffron quality using NIR-based predictive modeling regardless of the situation. It should be pointed out that research on saffron samples collected from different regions is required to further calibrate the NIR method established in this study and increase the scope of the application of this method. However, these findings expand the toolbox available for the evaluation of saffron quality, indicating that the proposed model is fit for adoption as a method for the customs handling of large imports, laying a foundation for the study of other herbal materials featuring complex matrices.

## Figures and Tables

**Table 1:** LD, TCCC, and CP values and the assigned sets for predictive modeling.

Parameter	LD		TCCC		CP	
	Calibration Set	Prediction Set	Calibration Set	Prediction Set	Calibration Set	Prediction Set
Max (%)	11.2	10.7	19.5	19.5	14.8	13.4
Min (%)	6.4	7.5	10.0	10.0	7.0	7.0
Average (%)	8.65	8.53	14.86	15.2	10.54	10.41
Standard deviation	0.91	0.63	2.00	2.17	1.46	1.39
Variance	0.84	0.4	4.00	4.75	2.13	1.94

**Table 2:** Combination of preprocessing approaches and their performance for predictive modeling.

Pretreatment ^a^	Index	*R*	SEC	SECV	RPD
Method 1	LD	0.95	0.2994	0.3201	3.203
	TCCC	0.87	1.0289	1.1149	2.028
	CP	0.89	0.6924	0.7627	2.193
Method 2	LD	0.96	0.2542	0.2763	3.571
	TCCC	0.90	0.8687	0.9859	2.294
	CP	0.91	0.6213	0.6836	2.412
Method 3	LD	0.95	0.2962	0.3250	3.203
	TCCC	0.86	1.0299	1.1030	3.203
	CP	0.86	0.7617	0.8186	1.960

^a^ Method 1: standard normal variable transformation (SNV); detrended correction (DT); Savitzky–Golay smoothing; first-derivative Savitzky–Golay filtering; Mean centralization; Method 2: multivariate scattering correction (MSC); Savitzky–Golay smoothing; first-derivative Savitzky–Golay filtering; mean centralization; Method 3: Savitzky–Golay smoothing; first-derivative Savitzky–Golay filtering; mean centralization.

## Data Availability

Data are available within the article and Supplementary Materials.

## Supplementary Materials

The following supporting information can be downloaded at: https://www.mdpi.com/article/10.3390/molecules29173983/s1, Figure S1: Heating program map of LD of saffron samples; Table S1: Verification of TCCC results in 100 batches of saffron; Table S2: Verification of CP results in 100 batches of saffron; Table S3: Verification of LD results in 100 batches of saffron.
