# Supplementary material for: Evaluation of Saffron Quality Using Rapid Quantitative Inspection Technology with Near-Infrared Spectroscopy
Source: Molecules. 2024 Aug 23;29(17):3983. doi: 10.3390/molecules29173983 (PMC11396457; doi:10.3390/molecules29173983)
Supplement: Supplementary file 1 [file molecules-29-03983-s001.zip › molecules-3131877-supplementary.pdf]

Evaluation of Saffron Quality Using Rapid Quantitative Inspection Technology with Near-Infrared Spectroscopy

Ying Zhou[1], Han Zhang[2], Xiaohui Sheng[3], Rong Wang[1], Yao Yao[1], Qinglan Zhu[1], Ze Yi[1], Zhe Xu[4], Yi Wang[2], Cheng Zheng[1*], Yu Tang[2*]

1. NMPA Key Laboratory of Quality Evaluation of Traditional Chinese Medicine (Traditional Chinese Patent Medicine), Zhejiang Institute for Food and Drug Control, Hangzhou 310052, China
2. Pharmaceutical Informatics Institute, College of Pharmaceutical Sciences, Zhejiang University, Hangzhou 310058, China
3. Hangzhou Puyu Technology Development Co., Ltd. Hangzhou 310052, China
4. College of Pharmacy, Shenyang Pharmaceutical University, Benxi 117004, China

* Correspondence: chengzheng@zjyj.org.cn (C.Z.); yutang@zju.edu.cn (Y.T.)

Supplementary Information

Content

Supplemental Figures..3
Data processing steps of NIR spectra for quantitative analysis 3
Impact of different methods on the model's performance..4
Supplemental Tables 5
Table S1. Verification results of TCCC in 100 batches of saffron 5
Table S2. Verification results of CP in 100 batches of saffron..7
Table S3. Verification results of LD in 100 batches of saffron 10

Supplemental Figures

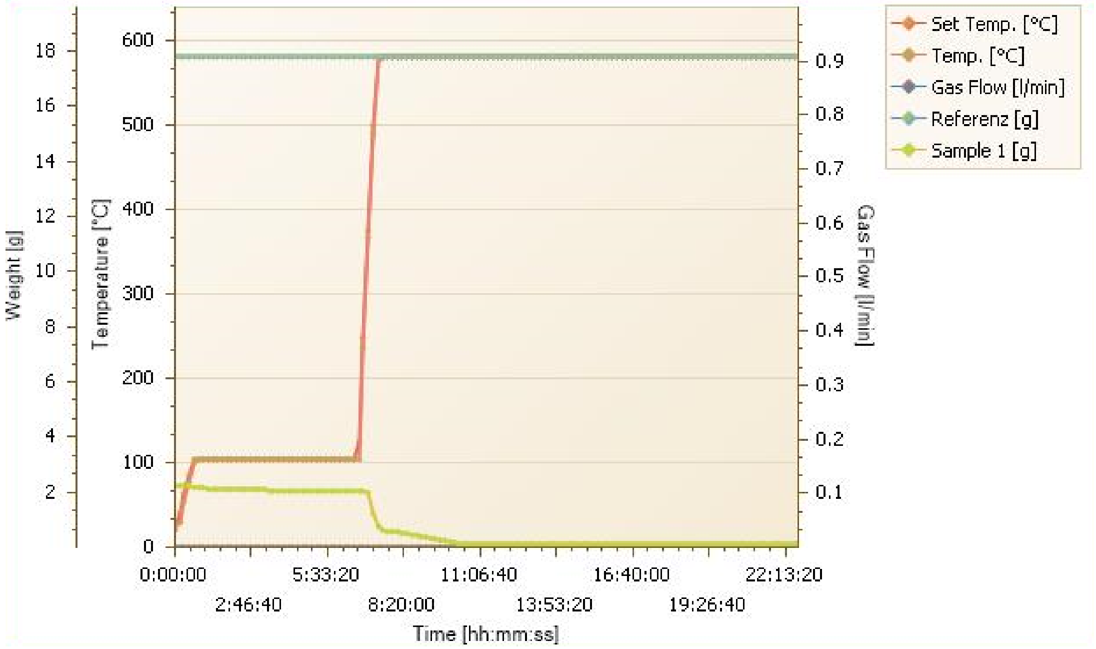

Figure S1. Heating program map of LD in saffron sample

Data processing steps of NIR spectra for quantitative analysis

In the RIMP software, the pretreatment algorithms for near-infrared raw spectra include methods such as Savitzky-Golay derivative, Savitzky-Golay smoothing, multiplicative scatter correction (MSC), standard normal variate transformation (SNV), and detrending correction (DT). One can freely choose the types of pretreatment methods and the processing sequence, but different pretreatment methods have a relatively large impact on the model results. We used the heuristic modeling function in the RIMP software to construct quantitative models between spectral data and the concentration values of LD, TCCC, and CP in saffron samples by the PLS (Partial Least Squares) method. The PLS method was adopted to establish models for each property.

First of all, the maximum principal factor of the PLS method, the number of partitions of the K value in the K-fold cross-validation, and the Mahalanobis distance threshold, student residual T value, and nearest neighbor distance threshold for judging abnormal samples could be set. Through the model calculation process, various parameters of the model such as SEC would be obtained at the end of the calculation.

Secondly, the confirmation principle of quantitative model includes the following:

1. It is generally believed that SECV <=1.2*SEC, indicating that the prediction effect of the model is good.
2. The closer R is to 1, the better the model effect, indicating that the correlation between the predicted value and the true value obtained by the model is very good.
3. An RPD value exceeding 2.0 demonstrates optimal prediction ability and model stability.
4. The above parameters SEC, SECVR and RPD should be integrated to judge the model effect and should not be used separately.

After the correction model is established, it can be used for the analysis of unknown samples. The near-infrared spectrum of the prediction set sample is input into the correction model, and then its composition and other properties are quantitatively detected and analyzed.

Impact of different methods on the model's performance

The correlation coefficient (R) represents the correlation between the values measured by the near infrared of the sample and those measured by the reference method. Under the condition of the same sample component content range, the R is closer to 1, and the model is better. The R values of LD in methods 1, 2 and 3 are basically the same, while the R values of TCCC and CP in method 2 are 0.90 and 0.91, respectively. Both values were higher than methods 1 and 3, indicating that the prediction model of method 2 was relatively superior.

SEC is the standard deviation of the residual difference between the values determined by NIR and the values determined by reference method in the calibration set. The SEC is smaller, the results of NIR analysis are more consistent with the results of chemical analysis. As can be seen from Table 2, the SEC values of LD, TCCC and CP in method 2 are 0.2542, 0.8687 and 0.6213, respectively. All of them are lower than SEC values in method 1 and Method 3, indicating that the results of NIR modeling by method 2 are the closest to the results of chemical analysis. Similarly, SECV is the standard deviation of the residual difference between the NIR and the reference values obtained during cross-validation during calibration. The accuracy of the model can be evaluated through SECV. The SECV is smaller, and the model effect is better. As can be seen from Table 2, the SECV values of LD, TCCC and CP in Method 2 are 0.2763, 0.9859 and 0.6836, respectively, which are all lower than the SECV values in method 1 and method 3, indicating that the accuracy of the model adopted in method 2 is good.

An RPD value demonstrates optimal prediction ability and model stability. It is generally believed that SECV <=1.2*SEC, RPD value exceeding 2.0, indicating that the prediction effect of the model is good. As shown in Table 2, the RPD values of LD, TCCC and CP in method 2 exceeding 2.0 are the largest among the three pretreatment methods. Based on the above results, method 2 is considered to be the optimal pretreatment method.

Supplemental Tables

Table S1. Verification results of TCCC in 100 batches of saffron

TCCC	actual measurement data/%	predictive value/%	Relative average deviation/%
sample 1	15.0	15.57	1.83
sample 2	16.0	16.20	0.57
sample 3	16.0	16.20	0.55
sample 4	17.0	16.05	2.87
sample 5	16.0	16.52	1.47
sample 6	16.0	16.32	0.92
sample 7	16.0	16.58	1.77
sample 8	17.6	16.81	2.29
sample 9	16.8	17.19	1.14
sample 10	17.0	16.93	0.21
sample 11	15.0	15.39	1.27
sample 12	15.0	15.10	0.34
sample 13	16.4	15.97	1.17
sample 14	15.0	14.78	0.71
sample 15	16.0	15.76	0.68
sample 16	15.0	15.36	1.19
sample 17	15.0	14.90	0.41
sample 18	16.0	16.12	0.37
sample 19	16.3	16.38	0.19
sample 20	16.5	15.63	2.70
sample 21	13.5	13.82	1.17
sample 22	14.0	14.66	2.31
sample 23	13.9	15.09	4.12
sample 24	14.0	14.29	1.04
sample 25	12.0	12.28	1.16
sample 26	14.0	15.44	4.89
sample 27	13.8	14.73	3.26
sample 28	11.0	10.74	1.21
sample 29	10.2	10.48	1.35
sample 30	12.0	11.81	0.78
sample 31	10.0	9.83	0.87
sample 32	13.0	13.10	0.39
sample 33	15.0	15.41	1.33
sample 34	10.1	10.93	3.96
sample 35	14.5	15.03	1.79
sample 36	11.9	13.51	6.34

sample 37	10.4	11.87	6.59
sample 38	11.7	13.15	5.82
sample 39	12.7	13.04	1.31
sample 40	14.2	14.39	0.66
sample 41	14.0	14.81	2.80
sample 42	14.6	14.26	1.18
sample 43	16.0	15.03	3.11
sample 44	12.0	13.57	6.13
sample 45	12.5	14.23	6.47
sample 46	14.0	14.61	2.14
sample 47	15.8	15.66	0.43
sample 48	16.4	15.67	2.27
sample 49	16.0	16.59	1.81
sample 50	13.1	13.64	2.03
sample 51	16.0	15.77	0.73
sample 52	17.0	16.43	1.70
sample 53	16.2	16.19	0.04
sample 54	16.0	16.54	1.67
sample 55	15.6	16.26	2.06
sample 56	17.5	17.21	0.84
sample 57	15.9	15.52	1.20
sample 58	14.2	13.87	1.17
sample 59	15.0	14.40	2.05
sample 60	15.0	14.05	3.27
sample 61	15.0	14.82	0.61
sample 62	15.2	14.70	1.66
sample 63	14.0	14.30	1.07
sample 64	14.1	14.88	2.68
sample 65	14.2	15.06	2.95
sample 66	14.0	14.72	2.50
sample 67	13.9	14.67	2.71
sample 68	12.0	14.96	10.98
sample 69	14.7	14.76	0.24
sample 70	14.6	14.53	0.24
sample 71	14.0	13.98	0.25
sample 72	13.3	13.27	0.26
sample 73	13.8	13.74	0.25
sample 74	14.1	14.07	0.25
sample 75	14.2	14.17	0.25
sample 76	14.0	13.89	0.25
sample 77	15.2	15.15	0.23
sample 78	14.4	14.34	0.24
sample 79	15.0	14.90	0.23
sample 80	14.5	14.40	0.24

sample 81	14.1	14.05	0.25
sample 82	17.6	18.50	2.50
sample 83	17.3	18.07	2.18
sample 84	19.0	18.27	1.96
sample 85	18.5	17.7	2.23
sample 86	18.2	18.1	0.19
sample 87	18.5	18.0	1.45
sample 88	19.5	18.9	1.67
sample 89	19.0	19.6	1.61
sample 90	19.0	18.2	2.10
sample 91	18.3	18.2	0.19
sample 92	18.1	18.6	1.36
sample 93	18.2	18.7	1.42
sample 94	18.3	18.0	0.75
sample 95	18.4	18.7	0.86
sample 96	18.5	18.2	0.84
sample 97	18.9	18.5	1.02
sample 98	18.0	18.4	0.98
sample 99	17.6	18.2	1.70
sample 100	16.8	17.0	0.51

Table S2. Verification results of CP in 100 batches of saffron

CP	actual measurement data/%	predictive value/%	Relative average deviation/%
sample 1	12.6	12.56	0.00
sample 2	10.6	10.60	0.09
sample 3	10.8	10.83	0.00
sample 4	11.0	11.05	0.23
sample 5	11.2	11.16	0.20
sample 6	11.3	11.14	0.72
sample 7	10.9	10.72	0.82
sample 8	10.8	10.87	0.32
sample 9	11.4	11.33	0.30
sample 10	11.0	11.11	0.50
sample 11	10.0	10.40	1.96
sample 12	10.3	10.17	0.65
sample 13	10.4	10.40	0.00
sample 14	9.5	9.41	0.45
sample 15	9.6	9.71	0.57
sample 16	9.3	9.34	0.19
sample 17	9.5	9.51	0.00

sample 18	10.6	10.56	0.00
sample 19	10.6	10.58	0.00
sample 20	10.5	10.49	0.00
sample 21	9.8	9.76	0.20
sample 22	10.0	9.98	0.09
sample 23	10.5	10.18	1.52
sample 24	10.2	10.09	0.56
sample 25	7.6	7.47	0.88
sample 26	9.6	10.36	3.81
sample 27	9.6	9.71	0.55
sample 28	7.3	7.17	0.88
sample 29	7.6	7.59	0.05
sample 30	7.9	8.16	1.65
sample 31	7.4	6.98	2.91
sample 32	7.8	8.37	3.55
sample 33	9.8	9.97	0.87
sample 34	7.0	7.89	5.98
sample 35	10.5	10.47	0.00
sample 36	8.2	9.35	6.56
sample 37	7.2	8.08	5.76
sample 38	7.7	9.22	8.96
sample 39	9.6	9.08	2.77
sample 40	11.8	10.85	4.21
sample 41	11.5	10.87	2.83
sample 42	10.9	9.85	5.06
sample 43	11.5	10.61	4.04
sample 44	9.1	9.89	4.17
sample 45	8.7	9.34	3.54
sample 46	8.8	9.21	2.27
sample 47	9.7	10.97	6.15
sample 48	10.0	10.58	2.83
sample 49	9.1	10.32	6.27
sample 50	9.9	9.95	0.00
sample 51	11.0	10.88	0.54
sample 52	11.2	11.26	0.26
sample 53	10.8	10.67	0.58
sample 54	11.5	11.28	0.97
sample 55	10.6	10.88	1.32
sample 56	11.2	11.58	1.68
sample 57	10.9	10.77	0.60
sample 58	10.0	9.87	0.68
sample 59	10.2	9.91	1.45
sample 60	10.1	9.96	0.71
sample 61	9.9	10.10	0.98

sample 62	10.0	9.78	1.11
sample 63	10.2	10.20	0.01
sample 64	10.2	10.43	1.13
sample 65	10.5	10.43	0.33
sample 66	10.2	10.15	0.26
sample 67	10.2	10.91	3.35
sample 68	10.2	10.70	2.40
sample 69	10.2	10.53	1.58
sample 70	10.0	9.75	1.28
sample 71	10.0	9.79	1.05
sample 72	10.0	9.83	0.86
sample 73	10.0	9.88	0.62
sample 74	10.0	9.88	0.63
sample 75	10.0	9.55	2.32
sample 76	10.2	9.76	2.18
sample 77	10.2	10.48	1.33
sample 78	10.2	10.63	2.04
sample 79	10.2	10.44	1.15
sample 80	10.2	10.08	0.58
sample 81	10.2	10.17	0.13
sample 82	11.7	11.82	0.50
sample 83	11.7	11.48	0.95
sample 84	11.7	11.44	1.13
sample 85	11.7	11.22	2.09
sample 86	11.7	11.86	0.67
sample 87	11.7	11.63	0.31
sample 88	11.8	12.77	3.95
sample 89	11.8	12.79	4.02
sample 90	11.8	11.79	0.05
sample 91	11.8	11.92	0.51
sample 92	11.8	12.19	1.61
sample 93	11.8	12.24	1.85
sample 94	13.4	12.56	3.24
sample 95	13.4	12.92	1.84
sample 96	13.4	12.75	2.50
sample 97	13.4	12.72	2.61
sample 98	13.4	13.24	0.58
sample 99	13.4	12.92	1.82
sample 100	11.1	11.32	0.19

Table S3. Verification results of LD in 100 batches of saffron

LD	actual measurement data/%	predictive value/%	Relative average deviation/%
sample 1	9.4	9.14	1.40
sample 2	9.0	9.01	0.03
sample 3	8.0	8.02	0.16
sample 4	8.0	7.91	0.56
sample 5	8.5	8.23	1.63
sample 6	8.2	8.22	0.09
sample 7	8.2	8.22	0.12
sample 8	8.1	8.03	0.42
sample 9	8.2	8.13	0.42
sample 10	8.2	8.18	0.13
sample 11	8.7	8.66	0.23
sample 12	8.2	8.23	0.15
sample 13	8.4	8.37	0.19
sample 14	8.4	8.46	0.34
sample 15	8.5	8.49	0.05
sample 16	8.4	8.36	0.25
sample 17	8.3	8.29	0.04
sample 18	8.2	8.19	0.06
sample 19	8.0	8.03	0.21
sample 20	8.5	8.56	0.35
sample 21	7.8	7.90	0.62
sample 22	7.6	7.44	1.04
sample 23	7.7	7.62	0.49
sample 24	7.7	7.72	0.16
sample 25	7.6	7.60	0.00
sample 26	7.5	7.60	0.64
sample 27	7.6	7.61	0.05
sample 28	9.1	8.71	2.17
sample 29	9.0	8.40	3.43
sample 30	8.6	8.45	0.86
sample 31	9.0	8.96	0.22
sample 32	8.9	8.52	2.18
sample 33	7.9	7.80	0.61
sample 34	9.2	9.33	0.69
sample 35	9.1	9.30	1.08
sample 36	9.5	9.58	0.41
sample 37	10.7	10.42	1.30
sample 38	9.8	9.44	1.85
sample 39	9.1	9.03	0.40

sample 40	9.1	8.95	0.83
sample 41	9.2	8.83	2.04
sample 42	9.4	9.29	0.58
sample 43	9.4	9.03	1.99
sample 44	9.5	8.93	3.08
sample 45	9.6	9.04	3.01
sample 46	9.5	9.10	2.12
sample 47	8.6	8.63	0.15
sample 48	8.2	8.25	0.31
sample 49	8.5	8.57	0.39
sample 50	9.0	9.13	0.73
sample 51	8.6	8.68	0.48
sample 52	8.4	8.40	0.01
sample 53	8.6	8.65	0.30
sample 54	8.6	8.64	0.22
sample 55	8.0	8.48	2.90
sample 56	8.4	8.40	0.01
sample 57	8.6	8.59	0.03
sample 58	9.2	8.91	1.59
sample 59	9.2	9.04	0.89
sample 60	8.9	8.86	0.05
sample 61	9.1	9.07	0.06
sample 62	9.2	9.13	0.37
sample 63	9.0	8.97	0.09
sample 64	9.1	9.21	0.62
sample 65	9.1	9.14	0.23
sample 66	9.1	9.05	0.30
sample 67	9.0	9.35	1.90
sample 68	9.1	9.17	0.40
sample 69	9.3	9.04	1.40
sample 70	8.9	8.99	0.51
sample 71	8.7	9.04	1.93
sample 72	8.7	8.81	0.64
sample 73	8.9	9.01	0.60
sample 74	9.2	9.18	0.13
sample 75	8.9	8.56	1.93
sample 76	8.5	9.05	3.13
sample 77	8.5	9.08	3.31
sample 78	8.8	8.84	0.24
sample 79	8.7	8.86	0.93
sample 80	8.7	8.77	0.41
sample 81	9.0	9.02	0.10
sample 82	7.6	7.70	0.64
sample 83	7.7	7.73	0.21

sample 84	7.6	7.57	0.22
sample 85	7.7	7.67	0.18
sample 86	7.8	7.80	0.03
sample 87	7.9	7.89	0.08
sample 88	7.5	7.72	1.47
sample 89	7.7	7.95	1.63
sample 90	7.8	7.77	0.16
sample 91	7.7	7.67	0.20
sample 92	7.6	7.56	0.24
sample 93	7.8	7.75	0.03
sample 94	8.0	8.26	1.58
sample 95	7.6	8.26	4.16
sample 96	8.1	8.07	0.16
sample 97	8.3	8.15	0.93
sample 98	8.4	8.38	0.10
sample 99	8.5	8.52	0.10
sample 100	7.6	7.976	2.42

NOTE: The actual measurement data was obtained according to the Chinese pharmacopoeia, and its significant number is reserved for one decimal place
